# Personal

**Published:** 1908-08

**Authors:** 


### PERSONAL.

---

DR. CHARLES R. BORZILLERI, of Buffalo, has been appointed treasurer of the Republican League of Clubs of the State of New York, succeeding John J. McWilliams, resigned. Dr. Borzilleri is a leading Italian physician in Buffalo, a man of accomplishment, and has recently opened one of the finest private hospitals in this region,—the Columbus Hospital—noticed in the July issue of the JOURNAL.

---

DR. JOHN DUGAN, of Albion, has been appointed health physician of that village for a term of four years, succeeding Dr. Frank G. Sherwood. Dr. Dugan is a graduate of the University of Buffalo, medical department, class of 1896.

---

DR. J. E. WALKER, of Hornell, N. Y., has been spending some months visiting the larger hospitals of Europe and, also, in making a special study of the methods employed at Bad Nauheim in the treatment of diseases of the heart. Dr. Walker returned about the middle of July and has resumed his work as superintendent of the Steuben Sanitarium at Hornell.

Dr. Augustus G. Pohlman, of Bloomington, Ind., who has for some time been junior professor of anatomy at Indiana University, recently has been promoted to a full professorship in the same chair.

---

Dr. Jane Wall Carroll, of Buffalo, who received the degree of bachelor of laws from the University of Buffalo last year, was created master of laws at the June commencement this year.

---

Dr. F. E. L. Brecht, of Buffalo, who with Mrs. Brecht and Miss Brecht has been spending several months in Europe, recently returned and has resumed his professional practice.

---

Dr. Walter C. G. Kirchner, of Saint Louis, superintendent and surgeon in charge of the Saint Louis municipal hospital, recently paid a short visit to Buffalo. Dr. Kirchner is making a tour of some of the northern cities while enjoying a few weeks vacation.

---

Dr. William P. Spratling, superintendent of the Craig colony for epileptics at Sonyea, N. Y., has resigned to take effect next November. Dr. Spratling is to accept the chair of nervous diseases and physiology in the College of Physicians and Surgeons at Baltimore, and will move to that city next fall.

---

Dr. George F. Cott, of Buffalo, accompanied by Mrs. Cott and their son DeLancey, sailed from Montreal, August 1, 1908, for a three months tour in Europe.

